# *Clematismae* (Ranunculaceae), a new species of C.sect.Meclatis from Xinjiang, China

**DOI:** 10.3897/phytokeys.114.31854

**Published:** 2019-02-14

**Authors:** Jian He, Ru-Dan Lyu, Min Yao, Lei Xie, Zong-Zong Yang

**Affiliations:** 1 Beijing Forestry University, 35 East Qinghua Rd. Haidian District, Beijing 100083, China Beijing Forestry University Beijing China; 2 Yang’s Herbarium, 728 North Xihuan Rd., Urumqi, Xinjiang 830011, China Yang’s Herbarium Urumqi China

**Keywords:** Anemoneae, Asia, Eudicots, Ranunculales, vine

## Abstract

*Clematismae* Z.Z.Yang & L.Xie, a new species of Ranunculaceae from Xinjiang, China, is described and illustrated. The new species is morphologically similar to *C.orientalis* and *C.glauca* but can be distinguished for being a less hairy plant (hairy in *C.orientalis*), often 2-ternate leaves (1–2-pinnate for *C.orientalis* and *C.glauca*), lanceolate to linear-lanceolate leaflets (elliptic or ovate in *C.glauca*), larger flowers (smaller flower in *C.orientalis*) and narrowly lanceolate sepals with acute to slightly attenuate apex (narrowly oblong sepals in *C.orientalis* and ovate to broadly lanceolate sepals in *C.glauca*). The new species is endemic to the southern slope of North Tianshan Mountain in Central Xinjiang. The conservation status of the species is also discussed.

## Introduction

*Clematis* L. is one of the three most widely distributed genera in Ranunculaceae (the other two being *Anemone* L. and *Ranunculus* L.; Ziman and Keener 1989), with approximately 300 species (Fang et al. 1980, Tamura 1987, 1995, Essig 1991, Johnson 1997, Grey-Wilson 2000, Wang and Li 2005, Miikeda et al. 2006, Xie et al. 2011, Lehtonen et al. 2016, Jiang et al. 2017). The taxonomy of *Clematis* has attracted much attention due to its great horticultural value. Historically, this large genus had been subdivided into many infrageneric groups using different taxonomic levels by different authors (Spach 1839, Prantl 1887, Tamura 1987, 1995, Johnson 1997, Grey-Wilson 2000, Wang and Li 2005).

Clematissect.Meclatis (Spach) Baillon also known as the *Orientalis* group (*sensu*Grey-Wilson 1989, 2000) is one of the taxonomically most difficult groups in the genus, with approximately a dozen yellow-flowered species that are widely distributed in Eurasia (Grey-Wilson 1989, Brandenburg 2000, Wang 2006). Species of C.sect.Meclatis are especially common in the highlands of central Asia and the Tibetan plateau. In his recent worldwide taxonomic revision of the section, Wang (2006) used leaf shape and colour, inflorescence type and position, sepal morphology and filament shape as key characteristics for species classification and accepted 13 species in C.sect.Meclatis. However, recent molecular phylogenetic analyses did not clearly resolve this morphologically defined section and showed that species of C.sect.Meclatis, C.sect.Fruticella and other species, like *C.barbellata* Edgew. and *C.pogonandra* Maxim., were nested together (Xie et al. 2011, Lehtonen et al. 2016). Many of C.sect.Meclatis species are widely distributed and also present a wide range of morphologic variation (e.g. *C.orientalis* L., *C.glauca* Willd., *C.tibetana* Kuntze and *C.tangutica* (Maxim.) Korsh.). There are also several narrowly distributed local species with very distinctive characteristics recognised by Wang (2006) (e.g. *C.sarezica* Ikonnikov, *C.caudigera* W.T. Wang and *C.corniculata* W.T. Wang). Recently, a new local species belonging to C.sect.Meclatis from Iran was also reported (Habibi et al. 2014).

During field investigations in Xinjiang, a distinctive population of *Clematis*, clearly belonging to sect. Meclatis, was discovered on the southern slope of North Tianshan Mountain. After carefully studying specimens of C.sect.Meclatis in Xinjiang and adjacent areas, we confirmed that this plant represents a distinctive taxonomic entity and thus describe it as a new species.

## Methods

Field investigations were conducted in the type locality and other areas in Xinjiang; specimens of C.sect.Meclatis were collected from Xinjiang and Gansu for morphological comparison. Furthermore, specimens of C.sect.Meclatis, deposited in PE, KUN, IBSC, BJFC, HIMC, IBK, NAS, XJA, XJBI, K, US and E were widely checked and evaluated using the relevant literature (Grey-Wilson 1989, Brandenburg 2000, Wang 2006). Morphological comparison and measurement of the specimens were carried out under a YKT5300 stereomicroscope. Newly collected specimens have been deposited in the herbaria of Beijing Forestry University (BJFC).

## Taxonomy

### Clematis mae

Taxon classificationPlantaeRanunculalesRanunculaceae

Z.Z. Yang & L. Xie sp. nov.

urn:lsid:ipni.org:names:77194986-1

Figs 1 , 2 , 3A–C

#### Diagnosis.

The new species is most similar to *C.orientalis* L. and *C.glauca* Willd. and it can be distinguished from the latter two species by the following combinations of characteristics. Plants of the new species are less hairy than *C.orientalis* and, in this respect, are similar to *C.glauca*. The leaves of the new species are often 2-ternate, with lanceolate to linear lanceolate leaflets. Its leaflets are larger than those of *C.orientalis*, but narrower than those of *C.glauca*. The flowers are also significantly larger than those of *C.orientalis* and slightly larger than those of *C.glauca*. The sepals of the new species are also less hairy than those of *C.orientalis* and similar to those of *C.glauca*. The shape of the sepal is lanceolate and the apex is acute to slightly attenuate. In *C.orientalis*, the sepals are often linear, oblong and reflexed. The sepals of *C.glauca* are often wider than those of the new species (Table 1, Fig. 3).

**Figure 1.**
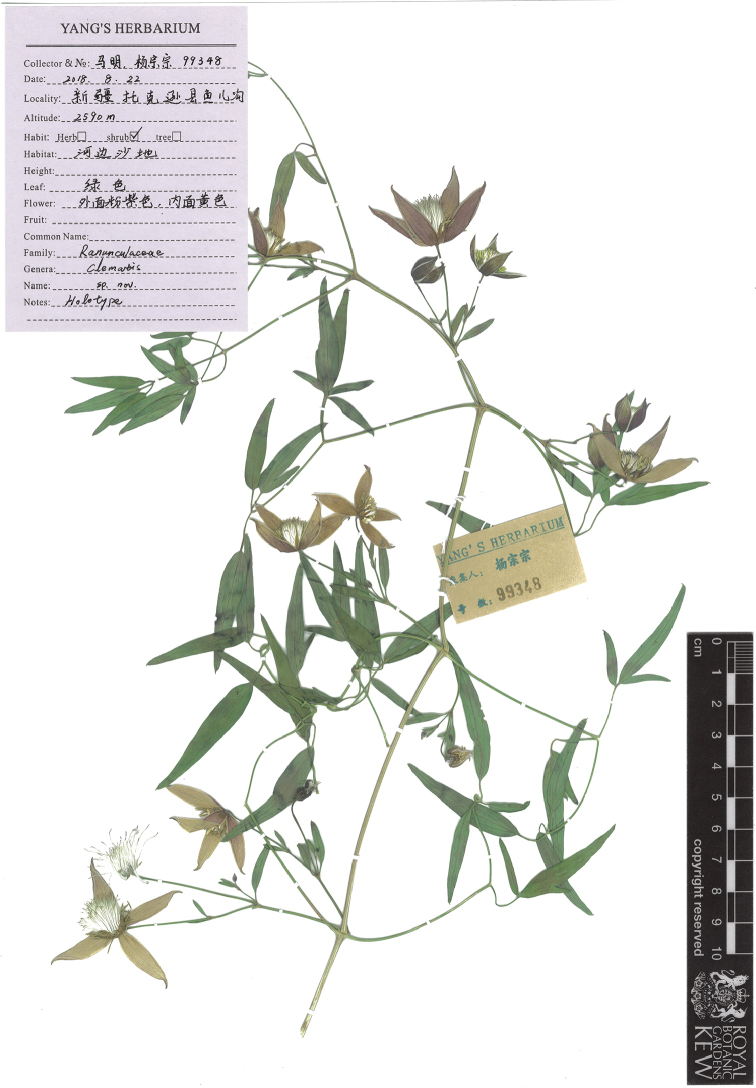
Holotype specimen (*M. Ma & Z.Z. Yang 99348*, deposited in BJFC) of the new species, *Clematismae* Z.Z.Yang & L.Xie, collected from Yuer gou, Toksun, Xinjiang, China.

**Figure 2.**
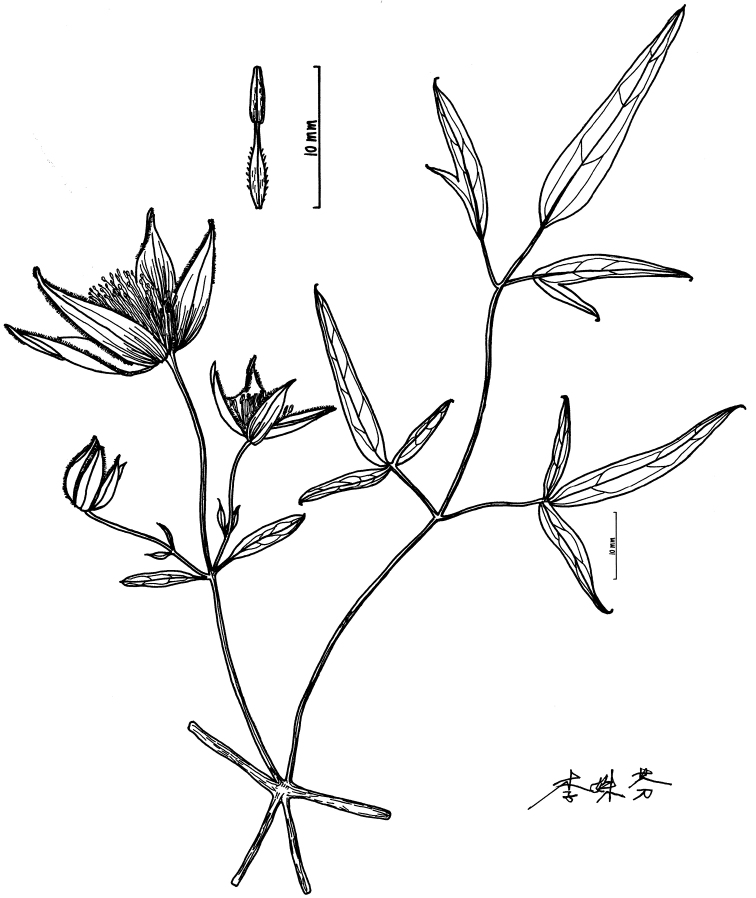
Illustration of *Clematismae* Z.Z.Yang & L.Xie. Drawn by S.F. Li

**Table 1.** Morphological comparison of the new species to two closely related species.

Species	* C. mae *	* C. orientalis *	* C. glauca *
Hairs on plant	Present but hard to see	Hairs often dense	Present but hard to see
Leaf	Bluish-green, pinnate to 2-ternate	Grey green, 1–2-pinnate	Bluish-green to green, 1–2-pinnate
Leaflet	Thick papery, always lanceolate to linear lanceolate, margin entire	Thick papery to subcoriaceous, highly variable, sometimes lanceolate, margin entire or 1–2 dentate	Papery to herbaceous, variable, often elliptic or ovate, margin entire
Inflorescence	1–3-flowered cyme	1-many-flowered cyme, often panicle like	1–7-many-flowered cyme
Bracteole	Entire	Entire	Sometimes 3-lobed
Flower	3.8–5.8 cm diam.	1.4–2.8 cm diam.	3.5–3.8 cm diam.
Sepal	Ascending, not reflexed	Spreading, reflexed	Ascending, not reflexed
Sepal color	Pinkish-purple outside	Yellow	Pinkish-purple or yellow outside
Sepal shape	Narrowly lanceolate	Often narrowly oblong	Narrowly ovate to elliptic
Inside sepal	puberulous	puberulous	glabrous or very sparsely puberulous
Stamen	7–12 mm long	5–9 mm long	7–14 mm long

**Figure 3.**
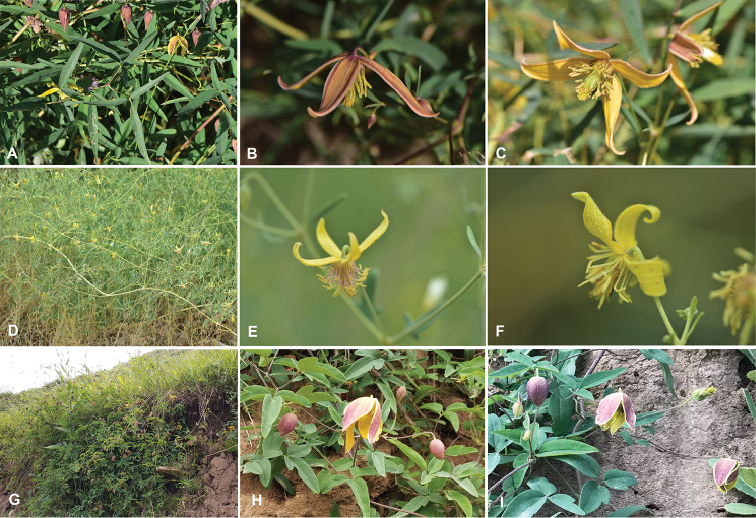
Field photographs of three closely related species of sect. Meclatis. **A–C***Clematismae* Z.Z.Yang & L.Xie. (photo taken by M. Ma & Z.Z. Yang) **A** Biternate leaf and flower buds **B** Ascending flower and its outside sepals **C** Flower inside **D–F***Clematisorientalis* L. (photo taken at Shihezi, Xinjiang, China, by Z.Z. Yang) **D** Habitat and plants of *C.orientalis***E** Flower showing spreading and reflexed sepals **F** Flower showing discernible hair on the inside sepals **G–I***Clematisglauca* Willd. (photo taken at Liancheng, Gansu, China, by J. He and L. Xie) **G** Habitat and plant in flower **H** Cyme and leaves **I** Flower and young fruit.

#### Type.

CHINA. Xinjiang: Toksun, Yuer gou, alt. 2590 m, sandy bank along the river, 22 Aug. 2018, fl, fr, *M. Ma & Z.Z. Yang 99348* (holotype: BJFC; isotypes: 3 ex BJFC).

#### Description.

Woody vine. Branches slender, shallowly 6 sulcate, very sparsely puberulous. Leaves pinnate to 2-ternate, very rarely ternate, up to 16 cm in length and 13 cm in width; living leaflets dry, green or sometimes grey-green, papyraceous to chartaceous, lanceolate to linear-lanceolate, 1.8–6.5 × 0.3–0.8 cm, glabrous on the upper side, sparsely puberulous on the lower side, usually 2–3-lobed to 2–3-sect, sometimes undivided, terminal lobe lanceolate to linear-lanceolate, 0.4–0.8 cm broad, margin entire, never dentate, midrib adaxially flat or slightly prominent, abaxially clearly prominent. Cymes all axillary, (1–2–)3–flowered; peduncles 2.5–3.0 cm long, slender; bracts petiolate, leaflet-like, up to 3.5 cm long; central flower of the 3 flowered cyme without bracteole, two lateral flowers bracteolate; bracteole petiolate and leaflet-like, up to 8 mm long. Flower 3.8–5.8 cm diam.; pedicel 2.8–5.2 cm long, puberulous, upper pedicel usually densely hairy. Sepals 4, pinkish-purple outside and yellow inside, ascending, lanceolate, 18–27 × 4–6.5 mm, puberulous on both surfaces, outside margin velutinous, apex acute to slightly attenuate. Stamens more than 30, 9–12 mm long; filaments lanceolate linear, widened in the lower part, pubescent; anthers linear to narrowly oblong, 3–4 mm long, glabrous, apex obtuse, minutely apiculate. Carpels numerous, up to 60 per flower; ovaries pubescent; styles 8–12 mm long, densely villous. Achenes laterally compressed, elliptic, ca. 3.9 × 2.1 mm, puberulous; persistent styles 6.5 cm long, plumose.

#### Specimens seen (paratypes).

CHINA. Xinjiang: Toksun, Yuer gou, alt. 2600 m, 22 Aug. 2018 (fl, fr), *M. Ma & Z.Z. Yang 99349, 99355* (paratypes: BJFC).

#### Phenology.

Flowering and fruiting time: July to September.

#### Distribution.

Only known from its type locality, Yuer gou, Toksun, Xinjiang, China.

#### Vernacular name.

Ming Tie Xian Lian (明铁线莲; new Chinese name)

#### Habitat and conservation status.

According to currently available data regarding *C.mae*, it occurs only in its type locality. The environment of the habitat is stable. We found about 200 individuals of the new species scattered along the river bank (elevation ca. 2500–2600 m) of Yuer Gou, Toksun Co. and we did not find individuals of this species outside this area. The herbarium investigation showed that several C.sect.Meclatis specimens were collected in Toksun Co., e.g. *AJ Li & JN Zhu 7288, 7335* (PE) and *QR Wang* et al. *4209* (PE); however, these specimens were collected at least 70 km from Yuer Gou and morphologically belong to *C.orientalis*. Based on currently available data, we considered the new species to be a local species endemic to a small area of Yuer Gou, Toksun Co. The open areas of the riverside, in which the new species occurs, may be threatened by settlements and agricultural activities. Therefore, we propose that the new species should be treated as Endangered (EN) in the International Union for Conservation of Nature (IUCN) categories system (IUCN 2012).

#### Etymology.

The species epithet is chosen in honour of the collector, Ms. Ma Ming, who first noticed this new species and guided the last author to collect specimens.

#### Taxonomic notes.

The new species clearly belongs to sect. Meclatis by its ascending sepals and pubescent and linear-lanceolate stamen filaments (Tamura 1995, Wang 2006) and is more similar to *C.glauca* than to *C.orientalis*. Observations of hairs, leaflets, flower size and shape and other characteristics clearly demonstrate that the new species is a taxonomic entity distinct from *C.orientalis*. *Clematisorientalis* is one of the most widely distributed species in its genus and has a wide range of morphological variation (Grey-Wilson 1989, Brandenburg 2000, Wang 2006). Both Grey-Wilson (1989) and Wang (2006) recognised seven varieties. The leaf shape of *C.orientalis* is strikingly variable (summarised by Grey-Wilson 1989) and may be similar to that of *C.mae*, but the leaves of *C.mae* are thinner than those of *C.orientalis*. Moreover, *C.orientalis* is often identified by its grey leaves, hairy stems and flowers and reflexed linear-oblong sepals. *Clematismae* from central Xinjiang is less hairy than *C.orientalis* and has larger flowers with ascending lanceolate sepals (Fig. 3). The hair and floral characteristics of the new species are somewhat similar to those of *C.glauca*, another widely distributed species with wider leaflets and sepals. In comparison with *C.orientalis*, *C.glauca* has less hairy stems and flowers, much wider leaflets and sepals and non-reflexed sepals. The primary differences between the new species and *C.glauca* are their leaflet shape, sepal shape and flower shape and size. Furthermore, the bracteoles of *C.glauca* are sometimes 3-lobed, which is never the case in *C.mae*.

#### Additional specimen examined

##### 

***Clematisorientalis* L., Sp. Pl. 1: 543. 1753.**

AFGHANISTAN. Kokcha-Tal, *D. Podlech 12732* (E).AZERBAIJAN. Caucasus, *Kolakovsky 1413* (MW). CHINA. Inner Mongolia, Ejina, *ZY Zhu & DS Wen 013* (HIMC); Gansu, Minqin, *YQ He 3332* (PE, WUK); Gansu, Jiuquan, *Qinghai-Gansu Exped. 2968* (PE), *ZJ Dong 270* (WUK). Xinjiang, Altay Shan, *RC Ching 2813* (PE); Xinjiang, Aqtau, *YC Wang Y172*, *Y180* (BJFC); Xinjiang, Gongliu, *Xinjiang Exped. Inst. Northwest Bot. 2667* (PE); Xinjiang, Hami, *RC Ching 122* (PE); Xinjiang, Hejing, Baluntai, *TY Zhou* et al. *651335* (NAS), *T Zhang* et al. *0443* (KUN); Xinjiang, Hetian, *Kelimu 106* (XJBI); Xinjiang, Korla, *AJ Li & JN Zhu 8641* (PE); Xinjiang, Qira, *Xinjiang Exped. 56-129* (PE); Xinjiang, Kunlun Shan, *ZQ Xie 25* (XJA); Xinjiang, Shanshan, *AJ Li & JN Zhu 6692* (PE); Xinjiang, Shihezi, *ZZ Yang 0857, 0858* (BJFC); Xinjiang, Tian Shan, *TN Liou 2689* (PE); Xinjiang, Toksun, *AJ Li & J N Zhu 7288* (PE), *QR Wang* et al., *4290* (PE); Xinjiang, Turpan, *ZM Zhang 294* (PE); Xinjiang, Ürümqi, *TN Liou 2891* (PE); Xinjiang, Yecheng, *Qinghai-Xizang Exped. 87-764* (PE), Xinjiang, Kashi, *Abulimit 258* (XJA); Xinjiang, Zhaosu, *Xinjiang Exped. Inst. Northwest Bot. 2593* (PE). IRAN. Khorasan, *Koelz 16822* (US). KAZAKHSTAN. Dzhambul, *Raikova 2915* (PE); Western Tianshan, *Mekerov 400* (PE); Issyk, *A. Regel 498* (K).MONGOLIA. South-western Mongolia, *Огуреева s.n.* (MW). RUSSIA. Dagestan, *Куликова s.n.* (MW). TURKEY. Tortum, *Davis 47565* (K); Oltu valley, *JC Archibald 8281* (E).TURKMENISTAN. Aschabad, *P. Sintenis 1055* (E). UZBEKISTAN. Taskent, *Ellas, Murray & Newcomba 9873* (PE).

***Clematisglauca* Willd., Berl. Baumz. 65. t. 4, fig. 1. 1796.**

CHINA. Gansu, Liancheng, *J He & L Xie 2018GS009* (BJFC), *RF Huang 2111* (HNWP); Gansu, Tianzhu, *RF Huang 2601* (HNWP). Qinghai, Qilian, *collector unknown 8532* (HNWP); Qinghai, Huzhu, *BZ Guo 25547* (HNWP). Xinjiang, Altay Shan, *RC Ching 2332* (PE); Xinjiang, Burqin, *YR Ling 74-1008* (PE); Xinjiang, Ili, *XY Li YL 96015* (XJBI); Xinjiang, Gongliu, *YC Wang Y171* (BJFC); Xinjiang, Korla, *AJ Li & J N Zhu 8642* (XJBI); Xinjiang, Ulastai, *KC Kuan 3816* (PE); Xinjiang, Wenquan, *Hoch & J. R. Chen 86-308* (PE). Xinjiang, Ürümqi, *RC Ching 628* (PE). RUSSIA. Altai Mountain, Chuya River, *Elias, Weber, Tomb & Krasnoborov 4411* (PE).

##### Key to the species of Clematissect.Meclatis

**Table:** 

1	Leaflet margin entire or with 1–2 teeth per side	**2**
–	Leaflet margin regularly denticulate, dentate, serrate or crenate	**9**
2	Flowers solitary, terminal or also in lateral, axillary cymes	**3**
–	Flowers usually in lateral, rarely also in terminal cymes, never solitary and terminal	**4**
3	Sepals inside glabrous, corniculate at apex	*** C. corniculata ***
–	Sepals inside puberulous, not corniculate at apex	*** C. tibetana ***
4	Lateral cyme with one flower, sepals brown-purple, apex conrniculate	*** C. sarezica ***
–	Cyme with 1–3-many flowers, sepals yellow or tinged with purple, apex not coniculate	**5**
5	Leaflet blades narrowly elliptic or narrowly ovate, sepal inside glabrous or very sparsely puberulous	*** C. glauca ***
–	Leaflet blades lanceolate or linear-lanceolate	**6**
6	Sepal inside glabrous	*** C. intricata ***
–	Sepal inside puberulous	**7**
7	Sepal outside glabrous	*** C. ladakhiana ***
–	Sepal puberulous on both surfaces	**8**
8	Leaves grey green, 1–2 pinnate; sepal oblong, yellow outside, reflexed	*** C. orientalis ***
–	Leaves bluish-green, often 2-ternate; sepal lanceolate, purple outside, not reflexed	*** C. mae ***
9	Sepals inside glabrous	**10**
–	Sepals inside puberulous	**11**
10	Leaf margin crenate	*** C. akebioides ***
–	Leaf margin dentate or denticulate	*** C. tangutica ***
11	Sepal with tail-like projection 3–6 mm long at apex; flowers only solitary and terminal, never in axillary cymes	*** C. caudigera ***
–	Flowers usually in lateral, rarely also in terminal cymes, never solitary and terminal	**12**
12	Sepals puberulous outside	*** C. hilariae ***
–	Sepals glabrous outside	**13**
13	Leaflets usually narrowly ovate or lanceolate, undivided, apex attenuate, margin serrate	*** C. serratifolia ***
–	Leaflets broadly ovate or ovate, 2–3-lobed, apex acute, margin irregularly dentate	*** C. zandaensis ***

## Supplementary Material

XML Treatment for Clematis mae
